# A fermented bean flour extract downregulates LOX-1, CHOP and ICAM-1 in HMEC-1 stimulated by ox-LDL

**DOI:** 10.1186/s11658-016-0015-z

**Published:** 2016-08-12

**Authors:** Morena Gabriele, Laura Pucci, Margherita La Marca, Daniela Lucchesi, Clara Maria Della Croce, Vincenzo Longo, Valter Lubrano

**Affiliations:** 1 National Research Council (CNR), Institute of Biology and Agricultural Biotechnology (IBBA), Pisa Unit, Research Area of Pisa, Via Moruzzi 1, 56124 Pisa, Italy; 2 Fondazione CNR/Regione Toscana G. Monasterio, Via Moruzzi 1, 56124 Pisa, Italy; 3 Department of Clinical and Experimental Medicine, Section of Metabolic Diseases, University of Pisa, Via Paradisa 2, 56124 Pisa, Italy

**Keywords:** Fermented *Phaseolus vulgaris* L, HMEC-1, Oxidative stress, Ox-LDL, ER stress, CHOP, LOX-1, ICAM-1, IL-6, ET-1

## Abstract

This study focused on an extract from fermented flour from the *Lady Joy* variety of the common bean *Phaseolus vulgaris*. The extract, *Lady Joy* lysate (Lys LJ), is enriched in antioxidant compounds during the fermentation. We assessed it for its protective effect on endothelial cells treated with oxidized-LDL (ox-LDL). The oxidative stress was determined by measuring the contents of thiobarbituric acid-reactive substances and reactive oxygen metabolites. ICAM-1, ET-1 and IL-6 concentrations were assessed using ELISA. LOX-1 and CHOP expression were analyzed using both quantitative RT-PCR and ELISA or western blotting. Ox-LDL treatment induced significant oxidative stress, which was strongly reduced by pre-treatment with the extract. The ox-LDL exposure significantly enhanced ICAM-1, IL-6 and ET-1 levels over basal levels. Lys LJ pre-treatment exerted an inhibitory effect on ox-LDL-induced endothelial activation with ICAM-1 levels comparable to those for the untreated cells. IL-6 and ET-1 production, although reduced, was still significantly higher than for the control. Both LOX-1 and CHOP expression were upregulated after ox-LDL exposure, but this effect was significantly decreased after Lys LJ pre-treatment. Lys LJ alone did not alter the ICAM-1, IL-6 and ET-1 concentrations or CHOP expression, but it did significantly lower the LOX-1 protein level. Our data suggest that Lys LJ is an effective antioxidant that is able to inhibit the oxidation process, but that it is only marginally active against inflammation and ET-1 production in HMEC-1 exposed to ox-LDL.

## Introduction

Atherosclerosis is one of the major causes of cardiovascular disease. Szmitko et al. [1] describe it as a progressive and dynamic disease arising from inflammatory injury and endothelial dysfunction. Together with inflammatory mediators, endothelial dysfunction may contribute to different stages of atherosclerosis, including lesion formation and progression, plaque rupture, hemorrhage and thrombosis, and it is an early hallmark of the condition [2–4].

Vascular homeostasis is characterized by a tightly regulated action on vascular tone, cellular adhesion, vascular smooth muscle migration and resistance to thrombosis. It arises from a balance between vasodilators and vasoconstrictors, pro- and antioxidants, and pro- and anti-inflammatory molecules [2, 5].

Endothelial dysfunction occurs due to decreased nitric oxide bioavailability and an increased level of endothelin-1 (ET-1), angiotensin II and oxidants, which contribute to a disequilibrium in endothelium-derived relaxing and contracting factors [4]. Endothelial activation arises from an altered expression of cell surface adhesion molecules, such as intracellular adhesion molecule (ICAM-1); vascular cell adhesion molecule (VCAM) and E-selectin; pro-inflammatory cytokines such as tumor necrosis factor alpha (TNF-α) and interleukin-6; and vasoactive mediators (prostaglandins, ET-1 and nitric oxide) [6]. ET-1 is one of the most potent vasoconstrictor peptides. It is synthesized and released mainly by the endothelial cells, and is also involved in the proliferation and hypertrophy of smooth muscle cells [7].

In the pathogenesis of atherosclerosis, oxidized low-density lipoproteins (ox-LDL) cause injury, activation and dysfunction of the endothelium via lectin-like oxidized low-density lipoprotein receptor-1 (LOX-1), which is overexpressed in both early and advanced atherosclerotic lesions [1]. Under conditions of hypertension, hypercholesterolemia and diabetes, all of which are pathological states characterized by chronic oxidative stress and high ox-LDL levels, LOX-1 is highly expressed in the blood vessels [1, 8]. Identified as the major receptor for ox-LDL uptake in the endothelial cells, LOX-1 has also been found in macrophages, platelets, cardiac myocytes and vascular smooth muscle cells [1, 9, 10]. Through LOX-1 upregulation, ox-LDL induces increased levels of intracellular reactive oxygen species (ROS), which contribute to transcription-dependent adhesion molecule synthesis and expression via the activation of nuclear factor-kB (NF-kB) [1, 11].

NF-kB is an oxidant-sensitive transcription factor that plays a key role in the expression of pro-inflammatory genes, including interleukin-6 (IL-6) [12], a multifunctional cytokine that is widely implicated in cardiovascular disease. IL-6 secretion is upregulated in response to inflammation, angiotensin II and oxidative stress [4, 12].

Over the last decade, endoplasmic reticulum (ER) stress, often referred to as the unfolded protein response (UPR), has been recognized as a relevant factor in the promotion of atherosclerosis. Ox-LDL has emerged as an inducer of ER stress signaling in cultured endothelial cells through a mechanism involving altered ER calcium metabolism [13]. Ox-LDL also induces endothelial cell apoptosis via the LOX-1-dependent ER stress pathway and through the activation of ER stress sensors (IRE1 and PERK) and related pathways, which contribute to upregulation of the expression of DNA damage-inducible transcript 3 (CHOP), an ER stress-responsive transcription factor with pro-apoptotic activity [14].

Recent intervention trials assayed the effect of components of the Mediterranean diet on endothelial function [15]. Epidemiological evidence suggests the existence of a relationship between improved endothelial function and the consumption of foods containing polyphenols in both healthy people and patients with cardiovascular disease [16, 17]. Noll et al. [18] also showed that daily intake of a red wine phenolic extract mainly composed of catechin and epicatechin had beneficial effects on plasma homocysteine levels and endothelial dysfunction biomarker expression in hyperhomocysteinemic mice. Furthermore, foodstuffs rich in procyanidin compounds, such as apple polyphenols and grape seed extracts, are reported to have an inhibitory effect on LOX-1 expression and ox-LDL binding to LOX-1 [19].

Common beans (*Phaseolus vulgaris* L.) are suggested to be nutraceutical due to their high content of bioactive compounds that improve endothelial function and exert anti-apoptotic, anti-aging, anti-tumor and anti-proliferative properties [20]. Cholesterol-lowering action and an inverse association between legume consumption and ischemic heart disease, coronary disease, type 2 diabetes and obesity have also been described [21, 22].

The recent increase in interest in fermented legumes as nutraceuticals with protective effects against cardiovascular diseases has led to an increase in their production and consumption [23]. Indeed, fermentation is now widely used to preserve and improve the texture, flavor and digestibility of several foods [24], including legumes, where it enhances the bioactive compound content and the antioxidant activity, decreasing non-nutritional factors [23].

As previously described by La Marca et al. [25], the fermentation of a flour derived from a genetically selected variety of *P. vulgaris* named *Lady Joy* yielded larger amounts of antioxidants, specifically polyphenols and flavonoids, compared to the unfermented flour. In this study, we investigated the effect of this fermented product (*Lady Joy* lysate) on the functional properties of human microvascular endothelial cells (HMEC-1) exposed to ox-LDL.

## Material and methods

### Plant material

The *Lady Joy* bean is a variety of *Phaseolus vulgaris* L. It is genetically devoid of the toxic constituent phytohemagglutinin and contains phaseolamin (an alpha-amylase inhibitor). Flour was produced by grinding the seeds, and the *Lady Joy* lysate (Lys LJ) was produced by fermenting and drying the flour. Lys LJ was extracted with distilled water, sonicated and centrifuged at 3500 rpm in a Jouan CR3i centrifuge for 10 min at 4 °C. The supernatant was collected, filtered (0.2 μm, VWR International PBI) and kept at 4 °C in the dark until use. The extraction was carried out in triplicate. The *Lady Joy* (LJ) flour and lysate compositions were detailed by La Marca et al. [25].

### LDL isolation and TBAR production

LDL isolation and oxidation were performed as previously described [26]. The degree of oxidation was determined using a colorimetric method (Cayman Chemical) by measuring thiobarbituric acid-reactive substances (TBARs) expressed as nmol of malondialdehyde (MDA) per mg of LDL protein according to the method described by Esterbauer and Cheeseman [27].

### Cell culture

The HMEC-1 line was obtained from the Centre for Disease Control. HMEC-1 cells retain morphological, phenotypic and functional characteristics of normal human microvascular endothelial cells. All of the media and medium supplements for cell culture were purchased from Sigma-Aldrich.

Cells were grown in 199 medium (M199) supplemented with 10 % fetal bovine serum (FBS), 1 % L-glutamine, 100 units/ml penicillin, 100 μg/ml streptomycin, 10 ng/ml epidermal growth factor (EGF) and 1 μg/ml hydrocortisone at 37 °C in a humidified 5 % CO_2_ incubator.

In order to evaluate the effect of Lys LJ extract on cell viability, HMEC-1 cells were incubated for 24 h with increasing doses of Lys LJ extract, corresponding to 0, 0.07, 0.7 and 1.4 mg/ml. The highest concentration devoid of toxic effects (i.e., with a cell viability of over 95 %) was used in further investigations.

Before each treatment, HMEC-1 were pre-incubated for 48 h with M199 without phenol red, containing antibiotics and 1 % FBS. Then, after 1 h pre-treatment in the presence or absence of 0.7 mg/ml of Lys LJ extract, HMEC-1 were stimulated for 24 h with or without 200 μg of apolipoprotein B (apo B) per ml of ox-LDL.

### Cell viability

The trypan blue exclusion test and the MTT assay were performed to evaluate cell viability. The trypan blue exclusion test was used to count the number of viable cells presented in a cell suspension. Cells were trypsinized, harvested and then 10 μl cell suspension was mixed with 10 μl 0.4 % trypan blue. Unstained (viable) and stained (nonviable) cells were counted separately in the hemacytometer.

The MTT assay measures mitochondrial activity in living cells. Briefly, cells were incubated with 100 μl MTT (Sigma; 1 mg/ml) for 2 h at 37 °C in 5 % CO_2_. Upon incubation, the medium was removed and the cells solubilized in 100 μl of 10 % DMSO/90 % isopropanol. The amount of the dye released from the cells was quantified by measuring the optical density at 540 nm using an Eti-System multiplate reader (Sorin Biomedica). The optical density directly correlates with the amount of metabolically active cells.

### Reactive oxygen metabolite production

Reactive oxygen metabolites (ROMs) were detected using a d-ROMs Kit (Diacron International) following the manufacturer’s protocol. The results were expressed as a percentage relative to the control.

### Quantitative RT-PCR

Total RNA was isolated from HMEC-1 using PureZOL RNA isolation reagent (Bio-Rad) and reverse-transcribed to cDNA using an iScript cDNA Synthesis Kit (Bio-Rad) following the manufacturer’s protocol. Quantitative PCR was performed using the SsoFast EvaGreen Supermix (Bio-Rad) in the StepOnePlus Real-Time PCR System (Applied Biosystems). Gene primers were designed using Beacon Designer Software (Premier Biosoft International) and were:

LOX-1 forward 5’-CCTTTGCCTGGGATTAGTAGT-3’, reverse 5’-GCTCTTGTGTTAGGAGGTCA-3’

CHOP forward 5’-GAGAGTGTTCAAGAAGGAAGTGTA-3’, reverse 5’-CCCGAAGGAGAAAGGCAAT-3’

β-actin forward 5’-GAGATGCGT-TGTTACAGGAAG-3’, reverse 5’-TGGACTTGGGAGAGGACT-3’

β-actin was used as the housekeeping gene. Samples were assayed in triplicate and the gene expression was calculated using the 2^−ΔΔCT^ relative quantification method.

### Immunoblot analysis

Western blot analysis was performed according to La Marca et al. [25]. The antibodies used were anti-CHOP (1:500) and goat anti-rabbit (1:5000; Santa Cruz Biotechnology, Inc.). Immunoreactive proteins were visualized with a chemiluminescence reaction kit (EuroClone), and bands deriving from three independent experiments were electronically scanned and quantified with ImageJ software.

### ELISA

The ICAM-1, ET-1 and IL-6 concentrations in the medium were measured using an ELISA kit from Cayman Chemical. The LOX-1 protein level was evaluated via ELISA with a kit from Aviscera Bioscience.

### Statistical analyses

Statistical analyses were performed using GraphPad Prism version 4.00 for Windows (GraphPad Software). Assays were carried out in triplicate and the results were expressed as the mean values ± SD. Differences between samples were analyzed via one-way analysis of variance (ANOVA) with Dunnett’s multiple comparison test. A *p* value lower than 0.05 is considered significantly different.

## Results

As described by La Marca et al. [25], Lys LJ contained a significantly higher level of polyphenols (3.8 ± 0.24 vs. 2.348 ± 0.05 mg GAE/g DW, *p* < 0.001) and flavonoids (2.1 ± 0.1 vs. 1.6 ± 0.1 mg CE/g DW, *p* < 0.01) compared to the equivalent unfermented flour. It also showed a better antioxidant activity, as evaluated using the ORAC assay (1233 ± 23 vs. 730 ± 30 μmol TE/100 g DW, *p* < 0.001). However, the flavonol concentration was reduced after fermentation (0.4 ± 0.1 and 1.6 ± 0.05 mg QE/g dw respectively, *p* < 0.0001) and anthocyanins were not detected. Therefore, we tested the effect of the fermented bean flour on human microvascular endothelial cells (HMEC-1) under oxidative conditions.

To identify the optimal treatment conditions and detect possible cytotoxic effects, we used a toxicity curve with 0–1.4 mg/ml as the range of concentrations of Lys LJ extract. The highest concentration that did not cause toxic effects (i.e., cell viability of over 95 % as determined using the Trypan Blue dye exclusion test and MTT assay) following 24 h exposure was used in further investigations (data not shown). As previously described by Lubrano et al. [28], we used 200 μg of apo B/ml of ox-LDL to induce oxidative stress. We investigated the effect of 24 h exposure to 200 μg/ml ox-LDL, following 1 h pre-treatment with 0.7 mg/ml Lys LJ extract on HMEC-1 cells.

**Fig. 1:**
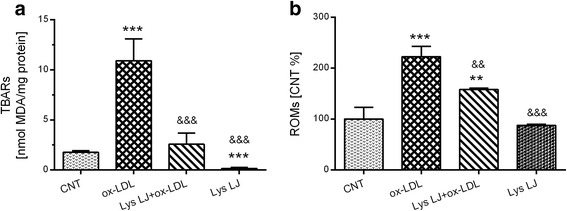
Quantification of the levels of TBARs (**a**) and reactive oxygen metabolites (**b**) in HMEC-1 cells pre-treated for 1 h with or without 0.7 mg/ml Lys LJ extract, then exposed for 24 h to 200 μg of apolipoprotein B (apo B) per ml of ox-LDL. Untreated cells were used as the control (CNT). Results are derived from triplicate determinations and expressed as means ± SD. ANOVA with Dunnett’s multiple comparison test. *Significantly different from the control: **p < 0.01; ***p < 0.001. &Significantly different from ox-LDL: &&*p* < 0.01; &&&*p* < 0.001

The degree of oxidation following ox-LDL treatment was quantified as the formation of TBARs, which are lipid peroxidation markers. Their level was significantly higher in ox-LDL-exposed cells (10.9 nmole MDA/mg protein) than in untreated cells (1.76 nmole/MDA/mg protein, ***p < 0.001 vs. the control; Fig. 1a). Moreover, Lys LJ pre-treatment significantly reduced TBAR formation arising from ox-LDL exposure (&&&*p* < 0.001) with values overlapping the control values (Fig. 1a). Otherwise, Lys LJ alone significantly decreased TBAR concentration compared to the values under basal conditions (***p < 0.001; Fig. 1a).

Additionally, as shown in Fig. 1b, we detected a medium level of reactive oxygen metabolites (ROMs), which are free radical-derived compounds. Their levels were significantly increased by ox-LDL exposure (***p < 0.001 vs. the control). Although the level was reduced by Lys LJ pre-treatment (&&*p* < 0.01 vs. ox-LDL) it was still significantly higher than the control (**p < 0.01).

**Fig. 2:**
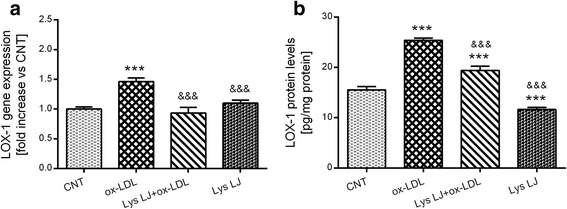
Quantitative RT-PCR (**a**) and ELISA (**b**) determination of LOX-1 expression in HMEC-1 cells pre-treated for 1 h with 0.7 mg/ml Lys LJ extract, then exposed for 24 h to 200 μg of apolipoprotein B (apo B) per ml of ox-LDL. Untreated cells were used as the control (CNT). Results are derived from triplicate determinations and expressed as means ± SD. ANOVA with Dunnett’s multiple comparison test. *Significantly different from the control: ***p < 0.001. &Significantly different from ox-LDL: &&&*p* < 0.001

**Fig. 3:**
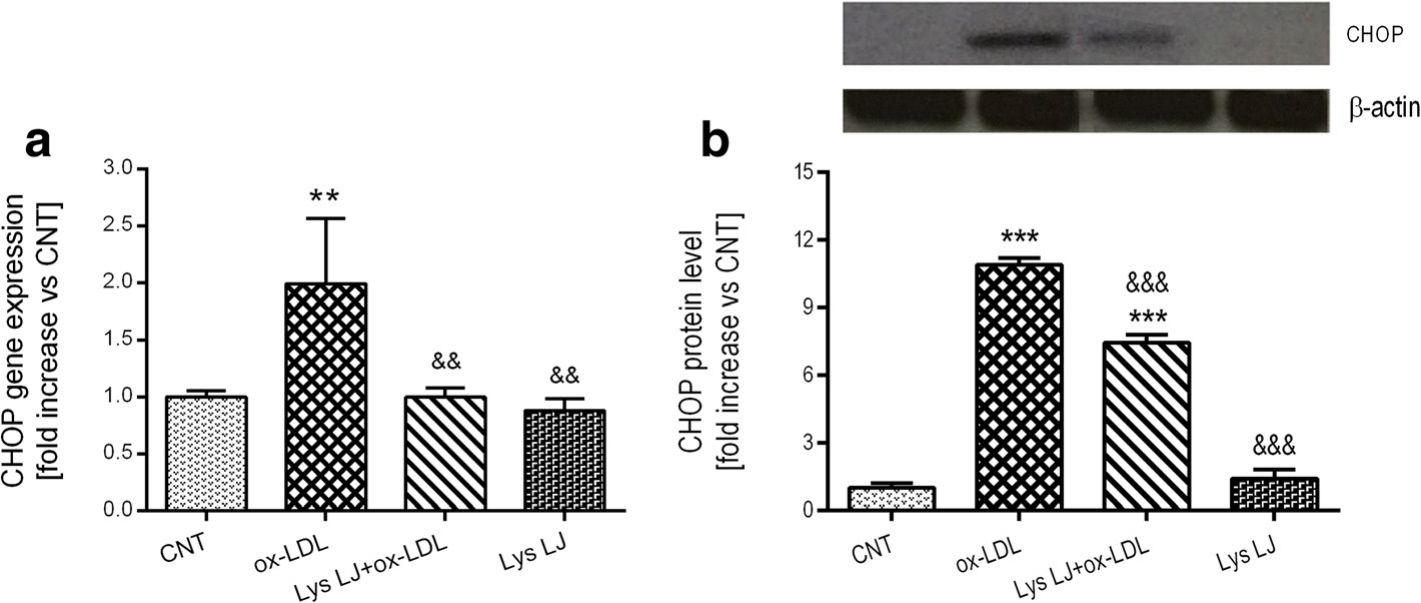
Quantitative RT-PCR (**a**) and western blot (**b**) analysis of CHOP expression in HMEC-1 pre-treated for 1 h with 0.7 mg/ml Lys LJ extract, then exposed for 24 h to 200 μg of apolipoprotein B (apo B) per ml of ox-LDL. Untreated cells were used as the control (CNT). The results are derived from triplicate determinations and expressed as means ± SD. ANOVA with Dunnett’s multiple comparison test. *Significantly different from the control: **p < 0.01; ***p < 0.001. &Significantly different from ox-LDL: &&*p* < 0.01; &&&*p* < 0.001

To identify the nutraceutical properties of Lys LJ, we tested its possible inhibitory effect on LOX-1 and CHOP expression and on the level of ICAM-1, IL-6 and ET-1 in the medium of endothelial cells following ox-LDL exposure. Quantitative PCR showed the expected significant upregulation of both LOX-1 (***p < 0.001 vs. the control) and CHOP (**p < 0.001 vs. the control) gene expression following ox-LDL treatment. This was strongly inhibited by Lys LJ pre-treatment (&&&*p* < 0.001 and &&*p* < 0.01 vs. ox-LDL, respectively). Conversely, Lys LJ treatment alone had no effect (Figs. 2a and 3a). These results were also confirmed at the protein level as shown in Figs. 2b and 3b. However, unlike with the gene expression, LOX-1 and CHOP proteins were reduced after Lys LJ pretreatment (&&&*p* < 0.001 vs. ox-LDL) but were still higher than in the control cells (***p < 0.001).

**Fig. 4:**
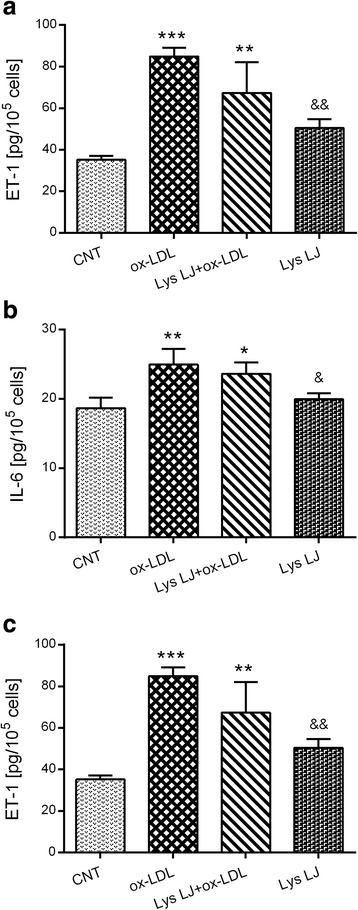
ELISA determination of ICAM-1 (**a**), IL-6 (**b**) and ET-1 (**c**) production in the medium of HMEC-1 cells pre-treated for 1 h with 0.7 mg/ml Lys LJ extract, then exposed for 24 h to 200 μg of apolipoprotein B (apo B) per ml of ox-LDL. Untreated cells were used as the control (CNT). Results are derived from triplicate determinations and expressed as means ± SD. ANOVA with Dunnett’s multiple comparison test. *Significantly different from the control: *p < 0.05; **p < 0.01; ***p < 0.001. &Significantly different from ox-LDL: &*p* < 0.05; &&*p* < 0.01; &&&*p* < 0.001

As shown in Fig. 4a–c, exposure of HMEC-1 to 200 μg/ml ox-LDL resulted in a significant upregulation of ICAM-1 (**p < 0.01), IL-6 (**p < 0.01) and ET-1 (***p < 0.001) concentrations in the medium compared to the untreated cells. Lys LJ pre-treatment exerted an inhibitory effect on ox-LDL endothelial activation with ICAM-1 levels overlapping the control values (Fig. 4a). Although Lys LJ pre-treatment of HMEC-1 cells exposed to ox-LDL reduced the levels of both IL-6 and ET-1, these levels remained significantly higher than in control cells (Fig. 4b and c). The exposure of cells to Lys LJ treatment alone produced no significant change in IL-6 or ET-1 levels.

## Discussion

Solid-state fermentation has recently been extensively used to improve the nutritional properties and enhance the bioactive compound content of certain foodstuffs, such as black beans [29]. As previously described by La Marca et al. [25], the fermentation process applied to *Lady Joy* bean flour increases the amount of antioxidants, in particular polyphenols and flavonoids, and significantly increases the antioxidant capacity of the bean lysate preparation compared to the unfermented flour. The same fermentation process applied to an organic grain flour named *Lisosan G* showed similar results with beneficial effects on HMEC-1 following ox-LDL exposure [30].

We previously showed improved human endothelial progenitor cell functions after Lys LJ pre-treatment in terms of cell viability, adhesion capacity and senescence rate both under basal conditions and following H_2_O_2_ exposure [31]. Furthermore, we investigated the effect of Lys LJ on human erythrocytes exposed to a radical generator, showing a good cellular antioxidant activity and a higher hemolysis inhibition following Lys LJ pre-treatment. We also described good anti-mutagenic and antioxidant activity following oxidative injury in the S*accharomyces cerevisiae* D7 strain pre-treated with Lys LJ extract [32].

To better define the nutraceutical properties of Lys LJ, we investigated the effect of pre-treatment with the extract in human microvascular endothelial cells. Overall, our findings show that Lys LJ pre-treatment could improve endothelial function otherwise impaired by ox-LDL exposure. In particular, Lys LJ pre-treatment could counteract the oxidation process arising from ox-LDL treatment and reduce the endothelial endogenous stress.

Thus, Lys LJ pre-treatment reduced the expression of LOX-1 at both the mRNA and protein levels. They were otherwise upregulated by exposure to lipoperoxides or free radical agents, which influences endothelial disfunction by promoting the generation of superoxide anions, the inhibition of nitric oxide production and the enhanced adhesion of monocytes to the endothelium [33, 34]. LOX-1 also contributes to inflammation and smooth muscle cell proliferation, leading to atheroma formation at multiple levels [35, 36]. The beneficial effects of Lys LJ were observed in these microvascular endothelial cells as the reduction of CHOP expression at both the mRNA and protein levels, in keeping with the earlier observations of La Marca et al. [25] with primary rat hepatocyte cultures exposed to H_2_O_2_.

The inhibitory effects of Lys LJ pre-treatment on the induction of ICAM-1 by ox-LDL treatment is noteworthy, where this pre-treatment completely inhibits the induction of ICAM-1 into the medium of HMEC-1 cells following ox-LDL treatment. These results are in keeping with a recent study in which flavonoids exhibited a good anti-inflammatory effect and reduced ICAM-1 expression in human aortic endothelial cells [37]. A similar effect was detected for HMEC-1 exposed to ox-LDL following pre-treatment with *Lisosan G* [38], which contains a significant amount of polyphenols [31]. Lys LJ pre-treatment was less effective with IL-6 and ET-1, where the increase in medium levels following ox-LDL treatmen was only partially reduced.

A recent study showed a synergic action of olive oil and wine polyphenols in the modulation of ox-LDL effects on oxidative stress [39]. Moreover, it has been demonstrated that the polyphenol fraction of extra virgin olive oil protected endothelial cells from free fatty acid-induced dysfunction by reversing ET-1 impairment [40]. Another recent paper showed that, among polyphenols, only resveratrol and quercetin were effective in decreasing H_2_O_2_-induced ET-1 overexpression in a dose-dependent manner in human umbilical vein endothelial cells (HUVEC). Although those authors did not provide a clear explanation for this, they suggested that these effects could depend upon the chemical structure and/or the antioxidant activity. Others reported that these two polyphenols showed the highest antioxidant capacities as measured by the ferric reducing antioxidant power assay [41].

We can therefore assume that this fermented bean flour may reduce oxidative stress by decreasing both lipoperoxides and free radicals. Our data also show an important role of Lys LJ in reducing endothelial adhesiveness, one of the main events in endothelial activation and plaque formation [42]. Indeed, adhesion of monocytes to the endothelium is one of the earliest cellular events in atherogenesis. Several mechanisms compete to recruit and promote cell–cell interaction between monocytes and endothelial cells [43].

## Conclusions

This study demonstrated the beneficial effects of a fermented bean flour lysate on human microvascular endothelial cells exposed to oxidative injury. Our results suggest that Lys LJ (*Lady Joy* lysate) is a strong antioxidant able to reduce the degree of oxidation, ER stress and endothelial adhesiveness induced by ox-LDL treatment. However, it is poorly active against inflammation and ET-1 induction resulting from pro-oxidant injury in human microvascular endothelial cells. The fermentation process applied to the bean flour is a useful procedure to enrich and ameliorate the nutritional properties of this legume, which might prove to be a useful nutraceutical for preventing cardiovascular diseases.

## Abbreviations

Apo B: apolipoprotein B
CHOP: C/EBP-homologous protein
ER: endoplasmic reticulum
EPCs: endothelial progenitors cells
ET-1: endothelin-1
EGF: epidermal growth factor
FBS: fetal bovine serum
HDL: high density lipoprotein
HMEC-1: human microvascular endothelial cells
HUVEC: human umbilical vein endothelial cells
IL-6: interleukin-6
ICAM-1: intracellular adhesion molecule
LOX-1: lectin-like oxidized low-density lipoprotein receptor-1
LJ: *Lady Joy*
LDL: low density lipoprotein
Lys LJ: *Lady Joy* lysate
MDA: malondialdehyde
M199: medium 199
NF-kB: nuclear factor-kB
Ox-LDL: oxidized-low density lipoprotein
ROS: reactive oxygen species
TBARs: thiobarbituric acid-reactive substances
TNF-α: tumor necrosis factor alpha
UPR: unfolded protein response
